# BMC Nephrology reviewer acknowledgement 2014

**DOI:** 10.1186/1471-2369-16-1

**Published:** 2015-01-23

**Authors:** Hayley Henderson

**Affiliations:** BioMed Central, Floor 6, 236 Gray’s Inn Road, London, WC1X 8HB UK

## Abstract

The editors of BMC Nephrology would like to thank all our reviewers who have contributed to the journal in Volume 15 (2014).

**Kevin Abbott**

USA

**Samar Abd Elhafeez**

Egypt

**Khaled Abdel-Kader**

USA

**Matthew Abramowitz**

USA

**Matteo Accetturo**

Italy

**Teresa Adragao**

Portugal

**Varun Agrawal**

USA

**Sabah Alharazy**

Malaysia

**John Allen**

Singapore

**Charles Alpers**

USA

**Michele Andreucci**

Italy

**Elio Antonucci**

Italy

**Michel Aparicio**

France

**Marta Arazzi**

Italy

**Christos Argyropoulos**

USA

**Masashi Asanoma**

Japan

**Brad Astor**

USA

**Alice Atzeni**

Italy

**Vincent Audard**

France

**Ahmad Taher Azar**

Egypt

**Marcelo Bacci**

Brazil

**Sunil Badve**

Australia

**Jyoti Baharani**

UK

**Matthew Bailey**

UK

**Phillippa Bailey**

UK

**Daniel Balkovetz**

USA

**Tanushree Banerjee**

USA

**Dongsik Bang**

Korea, South

**Irina Barash**

USA

**Carlo Basile**

Italy

**Rommel Bataclan**

Philippines

**Andrea Bauer**

Brazil

**Sahar Bayat**

France

**Justin Belcher**

USA

**Antonio Bellasi**

Italy

**Iddo Ben-Dov**

USA

**Laureline Berthelot**

France

**Francois Berthoux**

France

**Sebastjan Bevc**

Slovenia

**Markus Bitzer**

USA

**Natasa Bogavac-Stanojevic**

Serbia

**Piergiorgio Bolasco**

Italy

**Jason Bonomo**

USA

**Annalisa Botta**

Italy

**Josée Bouchard**

Canada

**L. Ebony Boulware**

USA

**Amarpali Brar**

USA

**Lauren Bresee**

Canada

**Sergey Brodsky**

USA

**Godela Brosnahan**

USA

**Steven Brunelli**

USA

**Bjoern Buchholz**

Germany

**Emmanuel Burdmann**

Brazil

**Nilka Burrows**

USA

**Gianfranca Cabiddu**

Italy

**Ronit Calderon-Margalit**

Israel

**Ruth Campbell**

USA

**Bernard Canaud**

France

**Angel Candela**

Spain

**Pietro Canetta**

USA

**Riccardo Cao**

Italy

**Alessandro Capitanini**

Italy

**Ben Caplin**

UK

**Cristina Capusa**

Romania

**Thomas Carroll**

USA

**Andre Carvalho**

Brazil

**Francesco Gaetano Casino**

Italy

**Giuseppe Castellano**

Italy

**Davide Catucci**

Italy

**Alejandro Chade**

USA

**Alex Chang**

USA

**Anthony Chang**

USA

**Hung-Chun Chen**

Taiwan

**Nan Chen**

China

**Tianxin Chen**

China

**Ying Chen**

USA

**Wai Leng Chow**

Singapore

**Byung Ha Chung**

Korea, South

**Mariann Churchwell**

USA

**Franco Citterio**

Italy

**Naomi Clyne**

Sweden

**Patrick Coates**

Australia

**Steven Coca**

USA

**Arthur Cohen**

USA

**Eric Cohen**

USA

**Jordana Cohen**

USA

**Joakim Cordtz**

Denmark

**Tom Cornelis**

Netherlands

**Frank Costantini**

USA

**Cécile Couchoud**

France

**Adrian Covic**

Romania

**Daniel Coyne**

USA

**Adamasco Cupisti**

Italy

**Marica Cutajar**

UK

**Wendy Dahl**

USA

**Aaron Dall**

USA

**Andrew Davenport**

UK

**John Davison**

UK

**Marc De Broe**

Belgium

**Mark De Caestecker**

USA

**Andreana De Mauri**

Italy

**Agostino De Pascale**

Italy

**Sophie De Seigneux**

Switzerland

**Anne-Emilie Decleves**

Belgium

**Claudia Del Corso**

Italy

**Dorella Del Prete**

Italy

**Lucia Del Vecchio**

Italy

**Pierre Delanaye**

Belgium

**Asterios Deligiannis**

Greece

**Sevag Demirjian**

USA

**Claire Den Hoedt**

Netherlands

**Patrice Deteix**

France

**Prasad Devarajan**

USA

**Patrick D'Haese**

Belgium

**Biagio Raffaele Di Iorio**

Italy

**Luca Di Lullo**

Italy

**Antonello Di Paolo**

Italy

**Clarissa Diamantidis**

USA

**Jun-Young Do**

Korea, South

**Mirela Dobre**

USA

**Mark Dockrell**

UK

**Kent Doi**

Japan

**Sonia Doi**

USA

**Tevfik Ecder**

Turkey

**Justin B. Echouffo-Tcheugui**

USA

**Charles Edelstein**

USA

**Philipp Eller**

Austria

**Ziad El-Zoghby**

USA

**F. Fevzi Ersoy**

Turkey

**Ciro Esposito**

Italy

**Vittoria Esposito**

Italy

**Michelle Estrella**

USA

**Paul Evans**

UK

**Colomba Falcone**

Italy

**Christopher Farmer**

UK

**Robert Fassett**

Australia

**Claude Ferec**

France

**Mariano Feriani**

Italy

**Hafedh Fessi**

France

**Fredric O Finkelstein**

USA

**Patrik Finne**

Finland

**Jennifer Flythe**

USA

**Daniele Focosi**

Italy

**Donald Fraser**

UK

**Simon Fraser**

UK

**Robert Frithiof**

Sweden

**Masafumi Fukagawa**

Japan

**Martina Gaggl**

Austria

**Ranil Gajanayaka**

USA

**Andrea Galassi**

Italy

**Daniel Gale**

UK

**Neetika Garg**

USA

**Karim Gariani**

Switzerland

**Giacomo Garibotto**

Italy

**Seyed Mansour**

Gatmiri Iran

**Joseph Gaut**

USA

**Adam Gaweda**

USA

**Duvuru Geetha**

USA

**Fernando Gerchman**

Brazil

**Berenice Gitomer**

USA

**Despoina Gkentzi**

Greece

**Glenda Gobe**

Australia

**Margarethe Goetz**

USA

**Sarah Goff**

USA

**Paraskevi Goggolidou**

UK

**David Goldfarb**

USA

**Matthew Griffin**

Ireland

**Konstadina Griva**

Singapore

**Jaap Groothoff**

Netherlands

**Fabrizio Grosjean**

Italy

**Jakob Gubensek**

Slovenia

**Encarna Guillen-Navarro**

Spain

**Zhiyong Guo**

China

**Orlando Gutierrez**

USA

**Mark Guy**

UK

**Andrew Hall**

Switzerland

**Bruce Hall**

Australia

**Garry Handelman**

USA

**Lisa Harrison-Bernard**

USA

**Donna H Harward**

USA

**Junichiro Hayano**

Japan

**John He**

USA

**Meian He**

China

**Martinez Ramirez Hector Ramon**

Mexico

**Hiddo Heerspink**

Netherlands

**Debra Higgins**

Ireland

**Nathan Hill**

UK

**Ewout Hoorn**

Netherlands

**Jennifer Hoponick Redmon**

USA

**Sabine Horn**

Austria

**Masato Hoshi**

USA

**Seyed Mohammadmehdi Hosseini Moghaddam**

Iran

**Raymond Hsu**

USA

**Weixin Hu**

China

**Shih-Han Huang**

Canada

**Michael Hultström**

Sweden

**Masimango Imani Mannix**

Congo, Democratic Republic

**Yoshitaka Isaka**

Japan

**Bernard Jaar**

USA

**Tazeen Jafar**

Singapore

**Deepika Jain**

USA

**Navin Jaipaul**

USA

**Matthew James**

Canada

**Aleksandar Jankovic**

Serbia

**Faical Jarraya**

Tunisia

**Vanita Jassal**

Canada

**Guillaume Jean**

France

**Robert Jenkins**

UK

**Julia S. Johansen**

Denmark

**Kirsten Johansen**

USA

**Heather Johnson**

USA

**Harald Jorstad**

Netherlands

**Veena Joshi**

Singapore

**Stephen Juraschek**

USA

**Claudine Jurkovitz**

USA

**Sahir Kalim**

USA

**Tomohiro Kamoda**

Japan

**Houda Kanoun**

Tunisia

**Vikas Kapil**

UK

**Andre Kaplan**

USA

**Ameet Karambelkar**

USA

**Riyaj Kasekar**

USA

**Mitsuhiro Kawano**

Japan

**Frieder Keller**

Germany

**Jessica Kendrick**

USA

**Eric Kerns**

USA

**Zeid Khitan**

USA

**In Joo Kim**

South Korea

**Won Kim**

Korea, South

**Seoyoung Kim**

USA

**Dimitrios Kiortsis**

Greece

**Alexander Kirsch**

Austria

**Krzysztof Kiryluk**

USA

**Hiroyuki Kobori**

USA

**Hirotaka Komaba**

Japan

**Judith Kooiman**

Netherlands

**Jeroen Kooman**

Netherlands

**Marion Koopman**

Netherlands

**Jay Koyner**

USA

**Andrea Kramer**

Netherlands

**Kalimuthu Krishnamoorthy**

USA

**Mei-Chuan Kuo**

Taiwan

**Ira Kurtz**

USA

**Mamoru Kusaka**

Japan

**Mi-Kyoung Kwak**

Korea, South

**Arjan Kwakernaak**

Netherlands

**Soon Hyo Kwon**

Korea, South

**Jean-Philippe Lafrance**

Canada

**Quirino Lai**

Italy

**Vijay Lapsia**

USA

**Thomas Larsen**

USA

**Alexandre Lautrette**

France

**Maurice Laville**

France

**Athina Lavrentieva**

Greece

**David Leaf**

USA

**Nelson Leung**

USA

**Moshe Levi**

USA

**Keith Levine**

USA

**Joshua Lewis**

Australia

**Karl Lhotta**

Austria

**Luyuan Li**

China

**Sophie Liabeuf**

France

**John Lieske**

USA

**Monica Limardo**

Italy

**Wender Lin**

Taiwan

**Yen-Chung Lin**

Taiwan

**Gregor Lindner**

Switzerland

**Kasia Lipska**

USA

**Francesco Locatelli**

Italy

**Carl Lombard**

South Africa

**David Long**

UK

**Joseph Longenekcer**

Kuwait

**Giuseppe Lucarelli**

Italy

**Giancarlo Lucchetti**

Brazil

**Randy Luciano**

USA

**Antonio Lupo**

Italy

**Francisco Maduell**

Spain

**Mitra Mahdavi-Mazdeh**

Iran

**Cherry Mammen**

Canada

**Filippo Mangione**

Italy

**Maria Martinez Cantarin**

USA

**Bashir Matata**

UK

**Vladimir Matchkov**

Denmark

**Sandro Mazzaferro**

Italy

**Marilda Mazzali**

Brazil

**William Mccumbee**

USA

**D. Olga Mcdaniel**

USA

**Susan Mclennan**

Australia

**Anita Mehrotra**

USA

**Kathan Mehta**

USA

**Bjorn Meijers**

Belgium

**Michal Melamed**

USA

**Cathy Mendelsohn**

USA

**David Mendelssohn**

Canada

**Shanthi Mendis**

Switzerland

**Robert Menzies**

UK

**Piergiorgio Messa**

Italy

**Wieneke Michels**

USA

**Farzanehsadat Minoo**

Iran

**Roberto Minutolo**

Italy

**Dana Miskulin**

USA

**Brett Mitchell**

USA

**Sandip Mitra**

UK

**Orson Moe**

USA

**Marcus Moeller**

Germany

**Femke Mollema**

Netherlands

**Stig Mølsted**

Denmark

**Divya Monga**

USA

**Alessandro Montanelli**

Italy

**Rafique Moosa**

South Africa

**Olivier Moranne**

France

**Rosa Moyses**

Brazil

**Miguel Moyses Neto**

Brazil

**Shrikant Mulay**

Germany

**Luisa Murer**

Italy

**Corrado Murtas**

Italy

**Raghavan Murugan**

USA

**Micho Nagata**

Japan

**Farid Nakhoul**

Israel

**Sankar Navaneethan**

USA

**Ana Navas-Acien**

USA

**Guy Neild**

UK

**Craig Nelson**

Australia

**Giuseppe Stefano Netti**

Italy

**Hannes Neuwirt**

Austria

**David Nikolic-Paterson**

Australia

**Shinichi Nishi**

Japan

**Claudia Nold**

Australia

**Donal O'Donoghue**

UK

**Aghogho Odudu**

UK

**William Oetting**

USA

**Ann O'Hare**

USA

**Yasushi Ohashi**

Japan

**Ercan Ok**

Turkey

**Klaus Olgaard**

Denmark

**Alberto Ortiz**

Spain

**Alfonso Otero Gonzalez**

Spain

**Zeynep Birsin Özçakar**

Turkey

**Carlos Palant**

USA

**Antonello Pani**

Italy

**Vincenzo Panichi**

Italy

**Rulan Parekh**

Canada

**Jung Tak Park**

Korea, South

**Andreas Pasch**

Switzerland

**Thakor Patel**

USA

**Rachel Patzer**

USA

**Adriano Peris**

Italy

**Maria Picken**

USA

**Francesca Pistoia**

Italy

**Laura Plantinga**

USA

**Carlos Poli De Figueiredo**

Brazil

**Rafael Ponikvar**

Slovenia

**Roberto Pontremoli**

Italy

**Stefan Porubsky**

Germany

**Maurizio Postorino**

Italy

**Valentina Postorino**

Italy

**Hans Pottel**

Belgium

**Giuseppe Quintaliani**

Italy

**Wajeh Qunibi**

USA

**Jochen Raimann**

USA

**Luca Rampoldi**

Italy

**Panduranga Rao**

USA

**Hugh Rayner**

UK

**Elrashdy Redwan**

Saudi Arabia

**Kevin Regner**

USA

**Flavio Reis**

Portugal

**Kyle Richards**

USA

**Azra Rizwan**

Pakistan

**Michael Robson**

UK

**Dario Roccatello**

Italy

**Alexander Rosenkranz**

Austria

**Mitchell Rosner**

USA

**Michael Ross**

USA

**Dvora Rubinger**

Israel

**Rupam Ruchi**

USA

**Gill Rumsby**

UK

**Domenico Russo**

Italy

**Abraham Rutgers**

Netherlands

**Dong-Ryeol Ryu**

Korea, South

**Ernesto Sabath**

Mexico

**Takako Saeki**

Japan

**Marcus Saemann**

Austria

**Seyed Ehsan Saffari**

Iran

**Amrik Sahota**

USA

**Hiroyuki Sakurai**

Japan

**Fadi Salem**

USA

**Richard Sandford**

UK

**Rajiv Saran**

USA

**Patrick Saudan**

Switzerland

**Judy Savige**

Australia

**John Sayer**

UK

**Arzu Sayiner**

Turkey

**Francesco Paolo Schena**

Italy

**Rebecca Scherzer**

USA

**Gernot Schilcher**

Austria

**Georg Schlieper**

Germany

**Ute Scholl**

Germany

**Savino Sciascia**

Italy

**Antonio Seguro**

Brazil

**Nicholas Selby**

UK

**David Selewski**

USA

**Donal Sexton**

Ireland

**Tariq Shafi**

USA

**Sanjeev Shah**

USA

**Linda Shavit**

Israel

**Neil Sheerin**

UK

**Kanji Shishido**

Japan

**Sandra Silveiro**

Brazil

**Justin Silver**

Israel

**Ana Cristina Simoes E Silva**

Brazil

**Pravin Singhal**

USA

**Ranga Siriwardhana**

Sri Lanka

**Meghan Sise**

USA

**Itzchak Slotki**

Israel

**Jayne Smith-Palmer**

Switzerland

**Laura Smyth**

UK

**Anna Solini**

Italy

**Ronak Soni**

India

**Manish Sood**

Canada

**Clemente Sousa**

Portugal

**Kevin Sowinski**

USA

**Stephen Sozio**

USA

**Adrian Specogna**

Canada

**Marijn Speeckaert**

Belgium

**J David Spence**

Canada

**Bruce Spinowitz**

USA

**Austin Stack**

Ireland

**Constantinos J Stefanidis**

Greece

**Tomasz Stompor**

Poland

**Elani Streja**

USA

**Toshihiro Sugiura**

Japan

**Ernest Sumaili**

Congo, Democratic Republic

**Randall Sung**

USA

**Chanderdeep Tandon**

India

**Sydney C.W. Tang**

Hong Kong

**Adis Tasanarong**

Thailand

**Avelino Teixeira**

USA

**Karthik Tennankore**

Canada

**Charuhas Thakar**

USA

**Mae Thamer**

USA

**Nicola Thomas**

UK

**Charlotte Thomas-Hawkins**

USA

**Joji Tokita**

USA

**Ashita Tolwani**

USA

**Allison Tong**

Australia

**Massimo Torreggiani**

Italy

**Yusuke Tsukamoto**

Japan

**J. Kevin Tucker**

USA

**Kriang Tungsanga**

Thailand

**Delphine Tuot**

USA

**Shigehiko Uchino**

Japan

**Susumu Uda**

Japan

**Jaime Uribarri**

USA

**Johannes G. Van Der Hoeven**

Netherlands

**Karlijn Van Der Pant**

Netherlands

**Frank Van Der Sande**

Netherlands

**Harry Van Goor**

Netherlands

**Barbara Van Munster**

Netherlands

**Mariacristina Vecchio**

Italy

**Andrea Veltri**

Italy

**Francisco Veronese**

Brazil

**Rachel Vieux**

France

**Giuseppe Villa**

Italy

**Folkert Visser**

Netherlands

**Astrid Wahl**

Norway

**Ron Wald**

Canada

**Stephen Waldek**

UK

**Rebecca Walker**

UK

**Stephen Walsh**

UK

**Yin Wang**

USA

**Makoto Watanabe**

Japan

**Amy Waterman**

USA

**Steven Weisbord**

USA

**Catharina Wesseling**

Finland

**Katherine Wesseling-Perry**

USA

**Francis Wilson**

USA

**Dawn Wolfgram**

USA

**Keng Thye Woo**

Singapore

**Alan Wright**

UK

**Julie Wright-Nunes**

USA

**Christina Wyatt**

USA

**Gaoqiang Xie**

China

**Eishin Yaoita**

Japan

**Pia Yngman-Uhlin**

Sweden

**Eric Young**

USA

**Sue Yung**

China

**Stuart Yuspa**

USA

**Hong Zhang**

China

**Ying Zhang**

USA

**Carmine Zoccali**

Italy

**Li Zuo**

China

